# Assessment of the Efficiency of Hospitals Before and After the Implementation of Health Sector Evolution Plan in Iran Based on Pabon Lasso Model

**Published:** 2017-03

**Authors:** Ghobad MORADI, Bakhtiar PIROOZI, Hossein SAFARI, Nader ESMAIL NASAB, Amjad MOHAMADI BOLBANABAD, Arezoo YARI

**Affiliations:** 1. Social Determinants of Health Research Center, Kurdistan University of Medical Sciences, Sanandaj, Iran; 2. Dept. of Health Management and Economics, School of Public Health, Tehran University of Medical Sciences, Tehran, Iran; 3. Dept. of Health in Emergencies and Disasters, School of Public Health, Tehran University Medical of Sciences, Tehran, Iran

**Keywords:** Pabon lasso model, Hospital, Utilization, Health system reform, Iran

## Abstract

**Background:**

Pabon Lasso model was applied to assess the relative performance of hospitals affiliated to Kurdistan University of Medical Sciences (KUMS) before and after the implementation of Health Sector Evolution Plan (HSEP) in Iran.

**Methods:**

This cross-sectional study was carried out in 11 public hospitals affiliated to KUMS in 2015. Twelve months before and after the implementation of the first phase of HSEP, a checklist was used to collect data from computerized databases within the hospitals’ admission and discharge units. Pabon Lasso model includes three indices: bed turnover, bed occupancy ratio, and average length of stay.

**Results:**

Analysis of hospital performance showed an increase in mean of bed occupancy and turnover ratio, which changed from 65.40% and 86.22 times/year during 12 months before to 69.97% and 90.98 times/year during 12 months after HSEP, respectively. In line with Pabon Lasso model, before the implementation of HSEP, 27.27% and 36.36% of the hospitals were entirely efficient and inefficient, respectively, whilst after the implementation of HSEP, their condition changed to 18.18% and 27.27%, in order.

**Conclusion:**

Indicators of bed occupancy and turnover ratio had a 4% increase in the studied hospitals after the implementation of HSEP. Number of the hospitals in the efficient zone reduced because of the relative measurement of efficiency by Pabon Lasso model. Since more than 50% of the hospitals in the studied province have not yet reached their optimal bed occupancy ratio (more than 70%), short-term and suitable strategy for improving the efficiency is to stop further expansion of hospitals as well as developing the number of hospital beds.

## Introduction

In recent years, the health sector in most countries has faced with a significant increase in hospital costs (1). Part of the problems is due to the construction of new and modern hospitals without primary assessments to collect and analyze information about people’s demands in a geographic area; lack of need assessments in many cases has resulted in inefficient use of resources (2). Efficiency refers to a circumstance in which the maximum output is achieved using a specific amount of money spent. For example, it checks whether using a specific number of beds available in a hospital results in the maximum number of patients treated in the same hospital (3, 4).

Inefficiency in the allocation and use of resources is one of the fundamental problems in health systems of developing countries (5, 6). Public hospitals, as the largest operating unit of health systems in developing countries, spend more than 50% of the total budget of health care while 80% of these resources are spent in hospitals whose efficiency is not more than 50% of their capacity (5, 7, 8). This is due to a combination of factors related to the supply, such as the excessive construction of hospitals without prior need-assessment, and poor quality of services; it is also caused by demand-related factors such as financial, geographical and cultural barriers to people’s access. One of the main concerns of policy-makers and health system managers in these countries is to improve the efficiency and performance of hospitals. In order to increase the efficiency of these centers, they try to implement some reforms in the health sector (3, 6, 9).

Performance of hospitals can be evaluated using several approaches. The Pabon Lasso model has been proved to be one of the most useful tools for comparing performance of different hospitals or different wards within the same hospital (6, 7, 10, 11).

This model is a graphical method that utilizes three indicators (Bed Turnover, Bed Occupancy Ratio, and Average Length of Stay) concurrently to assess the relative performance of hospitals. “In this method, the occupancy ratio (horizontal axis) is plotted against the turnover ratio (the vertical axis), with vertical and horizontal lines dividing the diagram into four regions (6)”. “The horizontal and vertical demarcations represent the mean values of the turnover ratio and occupancy ratio (6)”.

An assessment based on only one of the capacity utilization ratios (bed turnover (BTO), bed occupancy ratio (BOR) and average length of stay (ALS)) may be imperfect and misleading. For example, bed occupancy ratio may be relatively high in the presence of unnecessarily high average length of stay that in turn are caused by factors such as poor nursing care and improper scheduling of diagnostic and therapeutic interventions. Thus, although the bed occupancy ratio may indicate a good level of capacity utilization, the fact is that such a phenomenon is due to underperformance/inefficiency of the hospital. Therefore, “to avoid such misleading conclusions, it becomes necessary to make use of all the three indicators simultaneously to have a better picture (6)”. To this end, the Pabon Lasso model is useful to analyze the performance of hospitals (11, 12).

Since the implementation of HSEP on May 5, 2014, studies on capacity utilization using the Pabon Lasso Model have not been conducted in Kurdistan Province.

The HSEP is a stepwise national plan. The first phase of HSEP includes multiple packages such as providing free basic health insurance to all uninsured Iranians, reducing out-of-pocket payments for inpatient services in the hospitals affiliated to Ministry of Health and Medical Education (MoHME), supporting the retention of physicians in underprivileged areas, and improving quality of care in the hospitals affiliated to MoHME through increasing specialists and improving quality of inpatient and outpatient services.

The implementation of HSEP will lead to people’s increased access and utilization of hospital services (especially for poor households and uninsured people); implementation of HSEP will increase the usage of inpatient services in the hospitals and will affect hospitals performance indicators consequently through reducing out-of-pocket costs and increasing the number of full-time physicians in hospitals affiliated to MoHME (13, 14).

This study was prospected to use Pabon Lasso Model to evaluate the performance of the hospitals affiliated to KUMS before and after the implementation of HSEP.

## Materials and Methods

This descriptive, cross-sectional study was carried out in 11 public hospitals (two teaching and nine non-teaching hospitals) affiliated to Kurdistan University of Medical Sciences namely: Be’sat and Tohid Hospitals in Sanandaj, Abne Sina hospital in Kamyaran, Fajr and Bu Ali hospitals in Mariwan, Emam hospital in Saqez, Emam Hossain hospital in Bijar, Sallahedin Ayubi hospital in Bane, Shahid Beheshti hospital in Qorve, Emam hospital in Divandare and Shohada hospital in Dehgolan, Iran. Twelve months before (From Apr 21, 2013, to Apr 20, 2014) and 12 months after (May 22, 2014, to May 21, 2015) the implementation of the first phase of HSEP, data was collected using a checklist.

A checklist was designed to collect data about number of active beds, bed occupancy ratio, number of admissions, and number of patients discharged.

The study was approved by the Ethics Committee of the Kurdistan University of Medical Sciences.

The data required for this study was extracted from computerized databases within the hospitals’ admission and discharge units. Capacity utilization ratios (BTO, BOR, and ALS) were calculated and Pabon Lasso diagram was drawn using SPSS 18 (Chicago, IL, USA). Kurdistan province is located in west of Iran and has a population of over 1500000 people as well as 10 cities (15).

## Results

All the studied hospitals, including two teaching and nine non-teaching hospitals, were public and affiliated to KUMS. Table 1 presents the data on the number of active beds, active bed days, occupied bed days, and the number of discharges during the study. There was a total of 1578.81 and 1581.31 active hospital beds in all of the studied hospitals before and after the implementation of HSEP, respectively. As this table shows, the performance of inpatient wards in the hospitals after the implementation of HSEP increased, and the occupied bed days increased from 376916 to 403907. Furthermore, hospitals had a wide variation in terms of size; their sizes ranged from 20.4 to 368.6 active beds.

**Table 1:** Data on the performance of inpatient wards in the studied hospitals before and after the implementation of HSEP on May 5, 2014

**City**	HID*	**Active beds**		**Hospital beds/1000 population**		**Active bed-days**		**Occupied bed-days**		**Discharges**	
		**Before**	**After**	**Before**	**After**	**Before**	**After**	**Before**	**After**	**Before**	**After**
Kamyaran	1	71.40	70.70	0.67	0.66	26070	25806	12062	11613	6080	5771
Divandare	2	72.20	72.20	0.87	0.86	26352	26352	15896	15470	7576	8046
Saqez	3	177.51	177.70	0.83	0.82	64782	64882	41736	46096	17020	19200
Mariwan	4	119.30	119.30	1.06	1.05	43554	43554	32457	33797	12888	15392
	5	61.20	61.20			22326	22326	14558	13448	6136	5761
Dehgolan	6	20.40	20.90	0.32	0.32	7442	7637	4194	5422	1255	1528
Bane	7	98.81	103.12	0.73	0.76	36054	37632	23609	25070	12193	13194
Bijar	8	100.22	103.82	1.05	1.08	36568	37890	19668	21538	7666	7825
Qorve	9	145.20	145.70	1.04	1.04	53000	53185	28375	30141	11887	12492
Sanandaj	10	365.81	368.61	1.56	1.53	133524	134542	96804	100425	33460	33401
	11	346.80	338.10			126570	123403	87557	100887	19970	21270
Mean of all Hs		1578.81	1581.31	1.05	1.04	576242	577209	376916	403907	136131	143880

* Hospital identifier

Table 2 summarizes the hospital capacity utilization measures and the location of hospitals in the Pabon Lasso diagrams for both periods of the study. In all of the studied hospitals, the mean of bed occupancy ratio increased from 65.40% before to 69.97% after the implementation of HSEP. In the period before the implementation of HSEP, the mean of bed turnover was 86.22 patients per bed/year and the hospital stay was 2.76 d, whereas in the period after HSEP, these indices changed to 90.98 patients per bed/year and 2.8 d, respectively.

**Table 2:** Hospitals capacity utilization measures in the studied hospitals during 12 months before and after the implementation of HSEP on May 5, 2014

**Hospital ID**	**Average (Standard deviation) Length of Stay (days)**		**Bed Turnover Ratio (patient per bed)**		**Bed Occupancy Ratio (Confidence interval)**		**Location in Pabon Lasso graph (zone)**	
	**Before**	**After**	**Before**	**After**	**Before**	**After**	**Before**	**After**
1	1.98(1.51)	2.01(1.52)	85.15	81.62	46.26 (34.69–57.83)	45.00 (33.46–56.54)	1	1
2	2.09(1.60)	1.92(1.59)	104.93	111.44	60.32 (49.04–71.60)	58.70 (47.34–70.06)	2	2
3	2.25(1.71)	2.40(1.73)	95.88	108.40	64.42 (57.38–71.46)	71.04 (64.37–77.71)	2	3
4	2.51(1.61)	2.19(1.60)	108.03	129.01	74.52 (66.70–82.34)	77.59 (70.11–85.07)	3	3
5	2.37(1.55)	2.33(152)	100.26	94.13	65.20 (53.27–77.13)	60.23 (47.97–72.49)	2	2
6	3.34(1.01)	3.50(1.03)	61.51	73.11	56.35 (34.83–77.78)	70.99 (51.30–90.68)	1	4
7	1.93(1.48)	1.90(1.48)	123.41	127.97	65.48 (56.11–74.85)	66.61 (57.31–75.91)	3	2
8	2.56(1.68)	2.75(1.70)	76.50	85.67	53.78 (44.02–63.54)	56.84 (47.14–66.54)	1	1
9	2.38(1.66)	2.41(1.66)	81.86	85.73	53.53 (45.42–61.64)	56.67 (48.61–64.73)	1	1
10	2.89(2.12)	3.00(2.10)	91.47	90.61	72.49 (67.91–77.07)	74.64 (70.18–79.10)	3	4
11	4.38(2.41)	4.74(2.45)	57.58	62.91	69.17 (64.31–74.03)	81.75 (77.68–85.82)	4	4
Mean of all hospitals	2.76(1.82)	2.80(1.84)	86.22	90.98	65.40 (63.05–67.75)	69.97 (67.71–72.23)	−	−

**Fig. 1:**
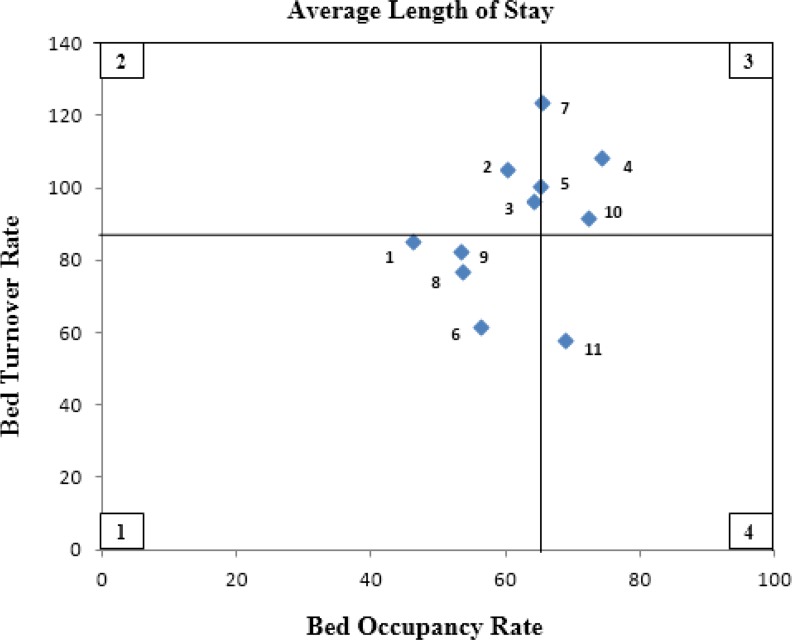
Pabon Lasso graph for public hospitals of Kurdistan University of Medical Sciences during 12 months before the implementation of HSEP on May 5, 2014

The location of hospitals in four zones of Pabon Lasso model illustrates during the period of study before the health system reform (Fig. 1). The horizontal and vertical lines indicate the mean values for bed occupancy ratio and bed turnover. Four hospitals (ID.1, 6, 8, 9) are located in zone one, indicating a large number of hospital beds relative to the existing demand; it also indicates the inefficient use of the resources available. There are three hospitals in zone 3 (ID. 4, 7, 10), indicating an acceptable degree of efficiency and an optimal level of performance. In addition, three hospitals (ID. 2, 3, 5) are located in zone 2 and one hospital (ID.11) in zone 4.

**Fig. 2:**
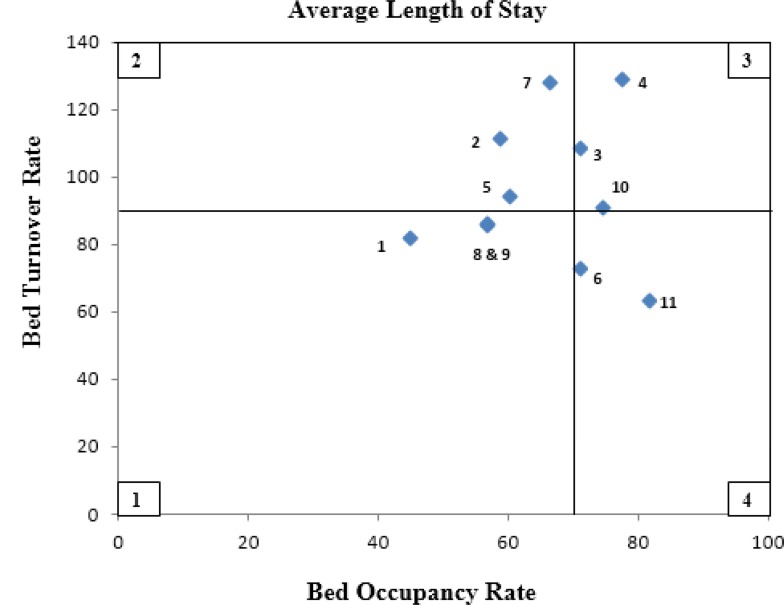
Pabon Lasso graph of public hospitals affiliated to Kurdistan University of Medical Sciences during 12 months after the implementation of HSEP on May 5, 2014

Fig. 2 illustrates the location of hospitals in four zones of Pabon Lasso model during the period of study after the implementation of HSEP. Two hospitals (ID. 4, 3) are located in the desirable zone of Pabon Lasso diagram (zone 3), while three hospitals (ID.1, 8, 9) are located in zone 1 which is the most undesirable situation characterized by low bed occupancy ratio and low turnover ratio. Moreover, three hospitals (ID. 2, 5, 7) are placed in zone 2 and three hospitals (ID. 6, 10, 11) in zone 4.

## Discussion

The average (SD) values for each of the three capacity utilization measures in the studied hospitals during 12 months before and after the first phase of HSEP were, respectively, as follows: ALS=2.76 (1.82) and 2.80(1.84) d, BOR=65.4% and 69.97%, BTO=86.22 and 90.98 patients per bed /year.

In the study period before HSEP, only two hospitals (18.18%) achieved the target value of BOR>70%, determined by the Iranian national standard (16, 17). While in the study period after HSEP, five hospitals (45.45%) had BOR more than 70%. However, most hospitals before (90.9%) and after (81.8%) the implementation of HSEP achieved the desired value of < 3.5 d that is determined by the Iranian national standard (16, 17).

The evidence indicates an increase in the use of hospitalization services during the period of study. In addition, in many hospitals (63.6%), bed turnover and occupancy ratio were higher after the implementation of HSEP compared to before its implementation. Nevertheless, despite the increase, the number of hospitals located in zone 3 of the Pabon Lasso graph reduced from three to two hospitals. This might be because the Pabon Lasso graph assesses the relative performance of hospitals and its horizontal and vertical lines indicate the mean values for bed occupancy ratio and bed turnover in all of the studied hospitals. For example, despite the increase in BOR (1.13%) and BTO (4.56 patients per bed/year), the location of Bane hospital (ID. 7) changed from zone 3 (before HSEP) to zone 2 after its implementation, because the observed increased values were less than the increased mean of values of BOR (4.57%) and BTO (4.76 patients per bed/year) in all of the studied hospitals. In a study in Eastern Azerbaijan province, Iran, 44.5% of the hospitals were entirely efficient (18).

As shown in Fig. 1 and 2, the number of hospitals located in zone 1, indicating poor performance and inefficient use of resources, declined from four to three after the health system reform.

The public hospital beds density per 1000 population in Kurdistan province was 1.05 and 1.04 before and after HSEP, respectively. Currently, Iran’s hospital beds density is 1.5 beds per 1000 population (private, public, and social security hospitals) while this number is 13.7 in Japan, 2.1 in Saudi Arabia, 2.5 in Turkey, and 5.3 in European region (19).

In the present study, bed density in cities of Kamyaran, Bijar, Qorve, and Dehgolan was lower than the value recommended for the size of the population; nevertheless, they were located in zone 1, indicating a surplus of hospital beds relative to the existing demand. However, this does not imply the presence of extra capacity relative to the demand. There may possibly be demand-side barriers of any type (e.g. financial, geographical, cultural, etc) or supply-side barriers (like poor quality services, lack of human resources or medical facilities) that negatively influence the utilization of hospital services. Currently, these cities do not need to set up new hospitals or add new beds; they should rather focus on removing barriers that make problems for the demand and supply sides of the hospital services.

One out of six hospitals (16.7%), three out of six hospitals (50%), and seven out of 18 public hospitals of Iranian Eastern Azerbaijan province (39%) were located in zone 1 (5, 7, 18).

In our study, three hospitals (ID. 2, 3, 5) before and three hospitals (ID. 2, 5, 7) after the health system reform lay in zone 2, which shows low BOR but high BTO ratio. Such hospitals do not need to increase the number of hospital beds and they should conduct studies to recognize problems that lead to low BOR. One out of 18 hospitals, and in Bahadori’s study, two hospitals (8.7%) lay in zone 2 (7).

Before the implementation of HSEP, only one hospital (ID. 11) was located in zone 4 while after HSEP three hospitals (ID. 6, 10, 11) were placed in zone 4; these hospitals were usually devoted to patients with chronic or serious illness or perhaps they had unnecessary long stay or were facing the under use of outpatient services. Tawhid and Besat hospitals (ID. 10, 11) are teaching hospitals and they have special words like burn, cardiology, cancer, NICU, and trauma wards that are intended to provide services for patients with chronic or serious illness. These hospitals are referral centers for critically ill patients in Kurdistan province. These could be the reasons for why they located in zone 4.

During the study period, Dehgolan general hospital (ID. 6) faced an increase of 14.64% and 11.6% in BOR and BTR, respectively, and it changed its position from zone 1 to zone 4. Although this change is a sign of improvement in performance, it is recommended to develop outpatient services in order to achieve optimum efficiency and utilize advanced medical equipment to improve BTR.

## Conclusion

Taking into consideration the overall performance of hospitals affiliated to KUMS, despite the increase in the average of bed occupancy and turnover ratio in the studied hospitals after the implementation of HSEP, the number of the hospitals in the efficient zone (zone 3) reduced because of the relative measurement of efficiency by Pabon Lasso model.

## Ethical considerations

Ethical issues (Including plagiarism, informed consent, misconduct, data fabrication and/or falsification, double publication and/or submission, redundancy, etc.) have been completely observed by the authors.

## References

[1] Yaisawarng S (2002). Performance measurement and resource allocation. Efficiency in the Public Sector. Springer US, pp. 61–81.

[2] Asefzadeh S (2003). Hospital Management and Research. 2nd ed Hadiseemrouz Publications Ghazvin.

[3] Roberts M Hsiao W Berman P Reich M (2008). Getting health reform right: a guide to improving performance and equity: Oxford university press London.

[4] Farrell MJ (1957). The measurement of productive efficiency. J R Stat Soc Ser A, 120( 3): 253– 90.

[5] Goshtasebi A Vahdaninia M Gorgipour R Samanpour A Maftoon F Farzadi F Ahmadi F (2009). Assessing hospital performance by the Pabon Lasso Model. Iran J Public Health, 38( 2): 119– 124.

[6] Asbu E Walker O Kirigia J Zawaira F Magombo F Zimpita P (2012). Assessing the efficiency of hospitals in Malawi: An application of the Pabón Lasso technique. Afr Health Monitor, 14: 25–33.

[7] Kalhor R Salehi A Keshavarz A Bastani P Orojloo P (2014). Assessing Hospital Performance in Iran Using the Pabon Lasso Model. Asia Pac J Health Manag, 9( 2): 77–82.

[8] Shepard D Hodgkin D Anthony Y (200). Analysis of hospital costs: a manual for managers. World Health Organization Geneva http://apps.who.int/iris/bitstream/10665/42197/1/9241545283.pdf

[9] Asefzadeh S Rezapour A (2005). Health Management. 2nd ed Hadise emrouz Ghazvin, Iran.

[10] Forootan S Arab M Hoseini M Khosravi B ( 2015). Determining the efficiency of social security hospitals of Tehran based on Pabon Lasso Model. J Health Adm, 18( 59): 7–18.

[11] Pabón L (1986). Evaluating hospital performance through simultaneous application of several indicators. Bull Pan Am Health Organ, 20( 4): 341–57. 3828621

[12] Younsi M ( 2014) Performance of Tunisian Public Hospitals: A Comparative Assessment Using the Pabón Lasso Model. Int J Hosp Res, 3( 4): 159–66.

[13] Moradi-Lakeh M Vosoogh-Moghaddam A (2015). Health Sector Evolution Plan in Iran; Equity and Sustainability Concerns. Int J Health Policy Manag, 4( 10): 637–40. 26673172 10.15171/ijhpm.2015.160 PMC4594102

[14] Iranian National Institute of Health Research (2014). Monitoring of Health Sector Evolution Plan. http://nihr.tums.ac.ir/wp-content/uploads/2015/04/file3.pdf

[15] Iran SCo (2012). Iran Statistical Yearbook. Statistical Centre of Iran Tehran, Iran

[16] Ministry of Health and Medical Education ( 2007). Standard guidelines and criteria of evaluation of public hospitals in the country. Ministry of Health and Medical Education Publication Tehran.

[17] Masoompour S Petramfar P Farhadi P Mahdaviazad H (2015). Five-year trend analysis of capacity utilization measures in a teaching hospital 2008–2012. Shiraz E-Med J, 16( 2): e21176.

[18] Mehrtak M Yusefzadeh H Jaafaripooyan E (2014). Pabon Lasso and Data Envelopment Analysis: A Complementary Approach to Hospital Performance Measurement. Glob J Health Sci, 6( 4): 107–16. 24999147 10.5539/gjhs.v6n4p107 PMC4825252

[19] World Health Organization (2014). World health statistics 2014. World Health Organization puplication Geneva http://www.who.int/gho/publications/world_health_statistics/2014/en/

